# The application of antimicrobial stewardship knowledge to nursing practice: A national survey of United Kingdom pre‐registration nursing students

**DOI:** 10.1111/jan.16195

**Published:** 2024-04-25

**Authors:** Molly Courtenay, Clare Hawker, Rose Gallagher, Enrique Castro‐Sanchez, Dinah J. Gould, Faten Al Salti, Jennifer Bate, Daniel Cooper, Rebecca Cooper, Rebecca Craig, Rebecca Dickinson, Debbie Fallon, Sharon Mcleod, Kate Morrow, Valerie Ness, Andrew Nichols, Sarah O'reilly, Sarah Partington, J. Claire Sevenoaks, Matthew Sunter, Jane Turner, Liz W. Underhill, Sarah L. Weaver

**Affiliations:** ^1^ School of Health Sciences Cardiff University Cardiff UK; ^2^ Infection Prevention and Control Royal College of Nursing London UK; ^3^ Brunel University Uxbridge UK; ^4^ Imperial College London UK; ^5^ University of Balearic Islands Spain; ^6^ Infection Control London UK; ^7^ Sheffield Hallam University College of Health & Wellbeing Sheffield UK; ^8^ School of Health, Science and Wellbeing Staffordshire University Shrewsbury UK; ^9^ School of Nursing and Advanced Practice Liverpool John Moore University Liverpool UK; ^10^ School of Health Sciences Medical School, University of Nottingham Nottingham UK; ^11^ School of Sport and Health Sciences University of Brighton Brighton UK; ^12^ School of Healthcare University of Leeds UK; ^13^ Manchester University Manchester UK; ^14^ Birmingham City University Birmingham UK; ^15^ School of Health and Social Work University of Hertfordshire Hatfield UK; ^16^ Department of Nursing and Community Health Glasgow Caledonian University Glasgow UK; ^17^ School of Nursing and Midwifery Plymouth University Plymouth UK; ^18^ Department of Health Sciences University of York UK; ^19^ School of Nursing and Healthcare Leadership Bradford University Bradford UK; ^20^ School of Health Sciences University of Greenwich London UK; ^21^ Robert Gordon University School of Nursing, Midwifery and Paramedic Practice Aberdeen UK; ^22^ School of Nursing and Midwifery University of Derby Chesterfield UK; ^23^ School of Nursing and Midwifery University of Hull Hull UK; ^24^ Three Counties School of Nursing and Midwifery University of Worcester Worcester UK

**Keywords:** biological subjects, nurse education, nurse roles, quantitative approaches

## Abstract

**Aim:**

To assess student nurses understanding and skills in the application of antimicrobial stewardship knowledge to practice.

**Design:**

Quantitative.

**Methods:**

Cross‐sectional survey.

**Results:**

Five hundred and twenty three student nurses responded across 23 UK universities. Although students felt prepared in competencies in infection prevention and control, patient‐centred care and interprofessional collaborative practice, they felt less prepared in competencies in which microbiological knowledge, prescribing and its effect on antimicrobial stewardship is required. Problem‐based learning, activities in the clinical setting and face‐to‐face teaching were identified as the preferred modes of education delivery. Those who had shared antimicrobial stewardship teaching with students from other professions reported the benefits to include a broader understanding of antimicrobial stewardship, an understanding of the roles of others in antimicrobial stewardship and improved interprofessional working.

**Conclusion:**

There are gaps in student nurses' knowledge of the basic sciences associated with the antimicrobial stewardship activities in which nurses are involved, and a need to strengthen knowledge in pre‐registration nurse education programmes pertaining to antimicrobial management, specifically microbiology and antimicrobial regimes and effects on antimicrobial stewardship. Infection prevention and control, patient‐centred care and interprofessional collaborative practice are areas of antimicrobial stewardship in which student nurses feel prepared. Interprofessional education would help nurses and other members of the antimicrobial stewardship team clarify the role nurses can play in antimicrobial stewardship and therefore maximize their contribution to antimicrobial stewardship and antimicrobial management.

**Implications for the Profession:**

There is a need to strengthen knowledge from the basic sciences, specifically pertaining to antimicrobial management, in pre‐registration nurse education programmes.

**Patient or Public Contribution:**

No patient or public contribution.

**Impact:**

**What Problem Did the Study Address?**

Nurses must protect health through understanding and applying antimicrobial stewardship knowledge and skills (Nursing and Midwifery Council 2018); however, there is no research available that has investigated nurses understanding and skills of the basic sciences associated with the antimicrobial stewardship activities in which they are involved.

**What Were the Main Findings?**

There are gaps in student nurses' knowledge of the basic sciences (specifically microbiology and prescribing) associated with the antimicrobial stewardship activities in which nurses are involved. Problem‐based learning, and activities in the clinical setting, were reported as useful teaching methods, whereas online learning, was seen as less useful.

**Where and on Whom Will the Research Have an Impact?**

Pre‐registration nurse education programmes.

**Reporting Method:**

The relevant reporting method has been adhered to, that is, STROBE.

## 1 BACKGROUND

Antimicrobial‐resistant infections (including bacterial, viral, fungal and parasitic infections) are among the greatest threats to human health globally (Antimicrobial Resistance Collaborators [ARC], 2019; World Health Organisation [WHO], 2020). Antimicrobials (including antibiotics, antivirals, antifungals and antiparasitics) are used significantly more per capita (per person) than in previous decades (CDDEP, 2015; WHO, 2020), and their misuse and overuse have been associated with an increase in antimicrobial resistance (AMR) (Llor & Bjerrum, 2014). Longer illnesses, increased mortality, prolonged stays in hospital, loss of protection for patients undergoing operations and other invasive procedures, and increased healthcare costs are all direct consequences of infection with resistant microorganisms (WHO, 2018). In 2019, 4.95 million deaths globally were associated with AMR (i.e. where AMR played some role) and 1.27 million deaths were attributable to AMR alone. The overuse of antimicrobials during the COVID‐19 pandemic, has further contributed to the public health threat from AMR (Strathdee et al., 2020). The last entirely original class of antibiotics was discovered in the late 1980s and few new antibiotics are available (Plackett, 2020).

## 2 LITERATURE REVIEW

To ensure that current antimicrobial options remain viable, antimicrobial stewardship (AMS) (the safe and effective use of antimicrobials) programmes have been developed internationally (Okeah et al., 2021) with the aim to reduce the misuse and overuse of antimicrobials. International European Commission [EC] (EC, 2017; EFNA, 2017; HM Government, 2019a) and national (HM Government, 2019b; Okeah et al., 2021) literature acknowledges nurses as vital to AMS efforts, with nurses performing numerous functions that are integral to the success of AMS programmes (Olans et al., 2016, 2017). For example, on admission to hospital, nurses are responsible for triage and appropriate isolation (if applicable) including taking an accurate allergy history, swabs/screening samples and early and appropriate blood cultures. They participate in interpreting and actively monitor microbiology results, monitor antibiotic dosing, de‐escalation and patients response to antimicrobial therapy. They are central communicators and co‐ordinators of care (Olans et al., 2016, 2017). Nurses consistent presence in healthcare delivery places them in a pivotal position to positively influence antimicrobial management (Gotterson et al., 2021). Furthermore, increasing numbers of nurses are qualified to prescribe medicines, and many of these nurses prescribe antibiotics (Courtenay et al., 2023).

Basic science provides the essential building blocks to understand biological processes of living systems in relationship to health, disease, treatment and prevention (National Institute of Genomic Medical Sciences, 2020). Basic science principles support the delivery of nursing care (Wu & Mahoney, 2022) and are recognized as important to the pre‐registration nursing curriculum (Horiuchi‐Hirose et al., 2023; Shahzeydi et al., 2022); however, nurses lack understanding and skills in the application of these sciences to practice (Camak, 2016; Jiale et al., 2018). The current Standards of Proficiency for registered nurses in the United Kingdom stipulate that nurses must protect health through understanding and applying AMS knowledge and skills (Nursing and Midwifery Council [NMC], 2018). This is a requirement not mandated in previous NMC standards (NMC, 2010). However, there is uncertainty and variation in nurses' perceptions of their contribution to AMS as part of their wider role, and the associated knowledge from the basic sciences (Gotterson et al., 2021); therefore, the full potential of their contribution to AMS is unlikely to be realized. In response to this issue, international competencies have been designed to address the spectrum of AMS activities in which nurses are involved (Courtenay et al., 2019). These competencies comprise of six key domains: (infection prevention and control [IPC]; antimicrobials and antimicrobial resistance; the diagnosis of infection and use of antibiotics; antimicrobial prescribing practice; person‐centred care [PCC]; and interprofessional collaborative practice [ICP]) representing the knowledge, skills, attitudes and values required for effective AMS. Each domain features competency descriptors reflecting the level of experience of the learner and type of practice setting, essential for AMS practice (Courtenay & Castro‐Sanchez, 2020). However, knowledge from each of the six domains, taught across UK pre‐registration nurse education programmes, is inconsistent, with lectures and case studies cited as the main strategies used to deliver AMS content (Courtenay et al., 2021). Furthermore, nurses understanding and skills in the application of AMS knowledge to practice is unknown. This study was designed to answer the research question ‘what is the understanding of student nurses in regard to their skills in the application of AMS knowledge in practice?’

## 3 THE STUDY

The aim of the study described below was to assess student nurses understanding and skills in the application of AMS knowledge to practice.

The study objectives were to identify:

- The AMS knowledge and skills in which student nurses feel prepared.
- Whether this is consistent across student nurses from UK universities.
- The teaching and assessment methods perceived to be most useful by student nurses with regard to feeling better prepared in AMS.

## 4 METHODS

### 4.1 Questionnaire

The study design adopted a cross sectional survey. The survey instrument was informed by research by Courtenay et al. (2019, 2021). These researchers developed international AMS competencies for undergraduate nurse education (Courtenay et al., 2019), and used a national cross sectional survey to look at the delivery of these competencies in UK pre‐registration nurse education programmes (Courtenay et al., 2021). Section one collected information on the level of academic award (i.e. degree or masters level). Section two asked student nurses how well they perceived the pre‐registration nurse education programme enabled them for their future practice as a nurse according to the six domains and descriptors (i.e. IPC, antimicrobials and antimicrobial resistance, the diagnosis of infection and use of antibiotics, antimicrobial prescribing practice, PCC and ICP) representing AMS noted above by Courtenay et al. (2019). Perceptions were collected using a 5‐point Likert scale (1 = not able, 3 = sufficiently able and 5 = very able). Section three asked student nurses how useful they perceived the methods used to teach (i.e. online learning, blended learning, face‐to‐face taught sessions, lectures, case studies, student presentations, activities in the clinical setting, problem‐based learning, simulation or other virtual environment and e‐learning), and assess (i.e. assessment essays, OSCE's, student presentation, student portfolio, short or long answer examination questions multiple choice examination) AMS learning to be. Perceptions were collected using a 5‐point Likert scale (1 = not at all useful, 3 = somewhat useful and 5 = very useful) Section four asked student nurses whether they shared AMS learning with students from other professions, and if helpful, why.

The survey was delivered via an online tool developed especially for creating web surveys (Online Survey).

### 4.2 Study setting and recruitment

All final year pre‐registration student nurses are from 35 UK universities. Undergraduate students from all nursing fields in the United Kingdom (i.e. adult, children, mental health and learning disabilities) were eligible to participate, as well as all students on a postgraduate route to registration (i.e., those with a prior degree not in nursing). Some of the student nurses were on the revalidated programme (i.e. following the 2018 NMC standards) (NMC, 2018); others were on an outgoing programme.

Previous research (Courtenay et al., 2021), involved an exploration of the delivery of international AMS competencies within pre‐registration nurse education programmes. Participants in this work, comprised an expert group of nurse educators, the nurse antimicrobial stewardship group (NAG) (Courtenay et al., 2021), were involved in teaching AMS and were representing 35 of the 72 universities offering pre‐registration nurse education in the United Kingdom. All NAG members acted as gatekeepers to recruit student nurses to this study. Gatekeepers were provided with a short slide presentation (including information on the threat of AMR to health, a definition of AMS, the inclusion of AMS in the NMC Standards of Proficiency for Registered Nurses), to deliver to all final year student nurses within their university, prior to disseminating the link to the online survey. The return of responses was slow and the decision was made to share the link on social media, specifically the Royal College of Nursing (RCN) student Facebook page. The survey link was also sent out via Twitter by the RCN Professional Lead, Infection Prevention and Control, and retweeted by the team and the wider Twitter community.

### 4.3 Data collection

All NAG members (*n* = 35) were invited to a 1 hr videoconference meeting. During this meeting, the NAG expert group, were asked whether the questionnaire items appeared to measure what they were supposed to measure (face validity) and whether they considered that it covered all aspects of antimicrobial stewardship adequately (content validity). The NAG members agreed unanimously that face and content validity had been achieved. We did not assess other aspects of validity or examine reliability through testing the internal consistency of questionnaire items as these items were based on previous research (Courtenay et al., 2019, 2021) and had already undergone development and scrutiny by experts. The survey was piloted on one cohort of degree level final year pre‐registration nursing students from one UK university. All students in the cohort were invited to take part. Fifteen students volunteered to participate. Only small formatting changes were made to the survey. The results of the piloted questionnaires were not included in the analysis.

During the data collection period, two further videoconference meetings took place between NAG members and the core research team. The aim of these meetings were to provide a forum in which any issues or challenges NAG members might experience in disseminating the survey link and recruiting students to the study could be discussed. Each meeting was recorded and a link to the recording was sent to all NAG members immediately afterwards.

Weekly follow‐up reminder emails were sent to students via the NAG gatekeepers. Data collection took place initially between February 2022 and June 2022. Responses rate was low (*n* = 450) and so therefore, the survey was reopened in October 2022 until March 2023.

### 4.4 Data analysis

Quantitative data were summarized according to rates of agreement/disagreement with each statement. Measures of central tendency (means, median and mode) and level of dispersion (standard deviation, interquartile range) were calculated to represent participants' collective judgements (Courtenay et al., 2019; Shepherd et al., 2017; Taylor et al., 2016). Median scores and interquartile ranges (IQRs) were calculated for responses to each statement to characterize the response category above and below which 50% of the responses fell. IQRs forming the distance between the 25th and 75th percentiles were used to represent the spread of the data and assess level of consensus per statement. Responses where the median was ≤ 2 (high level of agreement that the statement is important) with a small IQR (≤ 1.5) were taken as key statements for which consensus has been achieved. Responses with a median score of more than or equal to 3.5, with a small IQR (≤ 1.5), were taken as statements that had reached consensus concerning lack of importance.

Content analysis (Grbich, 2013) was used to categorize the free‐text comments, and explore qualitative findings. This process involved initial identification of commonly occurring themes, representing the range of responses. Themes were then broken down into mutually exclusive and exhaustive categories, and responses were assigned to categories and coded. The frequency of different responses was then counted. This process was performed manually.

### 4.5 Ethical considerations

Research Ethics Committee approval for the study was sought by MC and provided by the School of Healthcare Sciences Research Governance and Ethics Committee, Cardiff University (Reference No. REC REC830). The participant information sheet (PIS) and consent form were provided online at the beginning of the survey. Student nurses were required to tick a box indicating that they had read the PIS and consented to take part. They were informed that participation was voluntary and that they could withdraw at any point, that responses were strictly confidential and that information collected from the questionnaire would be anonymized.

## 5 RESULTS

### 5.1 Degree or masters level programmes

Across the 35 universities, 523 student nurses responded from 23 universities. The majority of students, 491 (89%), reported being on a degree level programme and 62 (11%) on a masters level programme.

### 5.2 Preparedness in AMS knowledge

Table 1 describes the extent to which student nurses felt prepared in knowledge from each of the six domains and descriptors representing AMS. For competency descriptors within domains one and two (IPC, and antimicrobials and AMR), there were high levels of agreement for all descriptors with medians in the strong range of agreement (4 or 5 on the 5‐point Likert scale). The strength of agreement was also high (IQR ≤ 2).

**TABLE 1:** AMS domains and descriptors.

	Median	IQR
Domain one: Infection prevention and control		
I feel able to describe what a microorganism is	4	2
2 I feel able to describe the different types of organisms that may cause infections	4	2
3 I feel able to explain what an antimicrobial‐resistant organism is	4	2
4 I feel able to explain the ‘Chain of Infection’	4	2
5 I feel able to define the components required for infection transmission (i.e. presence of an organism, route of transmission of the organism from one person to another and a host who is susceptible to infection)	4	1
6 I feel able to list the routes of transmission of infectious organisms that is, contact, droplet and airborne routes	4	1
7 I feel able to present and recognize the characteristics of a susceptible host	4	2
8 I feel able to demonstrate an understanding of the importance of surveillance	4	2
9 I feel able to describe how vaccines can prevent infections in susceptible persons	4	1
10 I feel able to demonstrate the application of standard precautions in healthcare environments	4	1
11 I feel able to apply appropriate policies/procedures and guidelines when collecting and handling specimens	4	1
12 I feel able to apply policies, procedures and guidelines relevant to infection control when presented with infection prevention and control cases and situations		
13 I feel able to implement occupational health practices that reduce the risk of infection (such as taking appropriate immunization or not coming to work when sick to ensure patient and other healthcare worker protection).	5	1
14 I feel able to understand that healthcare workers must be accountable and have an obligation to follow infection prevention and control protocols as part of their contract of employment	5	1
15 I feel able to act as a role model to healthcare workers and members of the public by adhering to infection prevention and control principles	5	1
16 I feel able to demonstrate knowledge and awareness of international/national strategies on infection prevention and control and antimicrobial resistance such as Global Action Plan for antimicrobial resistance and national recommendations, guidelines and legal requirements‐or equivalent	4	1
17 I feel able to understand the role of the environment in optimal infection prevention and control practices including hand hygiene and environmental cleaning	5	1
18 I feel able to enabling infection prevention and control self‐care for patients and family	5	1
Domain two: Antimicrobials and antimicrobial resistance		
I feel able to recognize the signs and symptoms of infection	5	1
2 I feel able to discuss how inappropriate antimicrobial use (including non‐adherence to treatment regime) may lead to antimicrobial resistance	4	1
3 I feel able to identify approaches to support optimal prescribing of antimicrobials	4	1
4 I feel able to recognize the importance of adequate specimen collection during relevant stages of antimicrobial use (i.e. prior/during antibiotic treatment)	4	2
5 I feel able to describe how to recognize the appropriate response to antimicrobial treatment and the main signs that demonstrate antimicrobial failures	4	1
Domain three: the diagnosis of infection and the use of antibiotics		
I feel able to explain how microbiology samples may aid diagnosis of infection	4	2
2 I feel able to describe how and demonstrate (following local procedures) the appropriate taking of samples	4	2
3 I feel able to interpret microbiology results/reports from the laboratory	3	2
4 I feel able to explain why self‐limiting bacterial or viral infections are unlikely to benefit from antimicrobials	3	1
5 I feel able to describe and demonstrate the self‐management strategies required to treat self‐limiting infections (i.e. analgesia/rest/fluids)	4	2
6 I feel able to understand the importance of following local antimicrobial policies (i.e. their development is based on local resistance patterns) and follow these policies in practice	4	2
7 I feel able to explain the importance of documenting the indications for an antimicrobial (i.e. the route by which it is administered, its duration, dose, dose interval and review date), in clinical notes and demonstrate this in practice	4	2
8 I feel able to demonstrate an understanding of the factors that need to be considered when choosing an antimicrobial (including site of infection and type of bacteria likely to cause an infection at a particular site)	4	1
9 I feel able to describe broad‐spectrum and narrow‐spectrum antimicrobials and the contribution of broad‐spectrum antimicrobials to antimicrobial resistance	3	2
10 I feel able to present and be able to recognize the common side effects associated with commonly administered antimicrobials	4	1
11 I feel able to demonstrate an understanding of why documenting a patient allergy to an antimicrobial is important	5	1
12 I feel able to explain why it is important to consider certain risk factors (such as renal function) in patients who receive an antimicrobial	4	1
13 I feel able to describe what is meant by delayed prescribing	3	2
14 I feel able to explain why it is essential that an accurate diagnosis of an allergy to an antimicrobial (such as penicillin) is based on history and laboratory tests	4	1
15 I feel able to demonstrate an understanding of the role of the nurse regarding quality and safety of antibiotic prescriptions	4	2
16 I feel able to demonstrate an awareness of laboratory results (i.e. culture and sensitivity) that demand prompt intervention	3	2
17 I feel able to recognize antimicrobials that should be preserved for treatment of specific infections, for example, carbapenemase‐producing Enterobacteriaceae (CPE) or colistin resistance or colistin‐resistant pathogens	3	1
Domain four: Antimicrobial prescribing practice		
I feel able to explain how to recognize and manage sepsis	5	1
2 I feel able to describe why it is important to use local guidelines to initiate prompt effective antimicrobial treatment in patients with life‐threatening infections	4	1
3 I feel able to describe why it is important to switch from intravenous antimicrobials to oral therapy	4	2
4 I feel able to describe how to switch from IV antimicrobials to oral therapy	4	2
5 I feel able to understand the appropriateness of antimicrobial administration models such as outpatient parenteral antimicrobial therapy (OPAT)	3	2
6 I feel able to demonstrate an understanding of the rationale and use of perioperative prophylactic antimicrobials to prevent surgical site infection	4	1
7 I feel able to discuss factors that can influence antimicrobial prescribing and the implications for antimicrobial stewardship programmes	3	2
8 I feel able to describe the national guidance on completion of a course of antimicrobials	4	2
9 I feel able to explain how you would identify the medicines with which antimicrobials can interact and why this is important	3	1
10 I feel able to describe the difference between empiric, targeted and prophylactic antimicrobial therapy	3	2
Domain five: Person‐centred care		
I feel able to support participation of patients/carers, as integral partners when planning/delivering their care surrounding antimicrobial treatment	4	2
2 I feel able to share information about antimicrobial treatment with patients/carers in a respectful manner and in such a way that is understandable, encourages discussion and enhances participation in decision‐making	4	2
3 I feel able to ensure that appropriate education and support surrounding antimicrobial treatment is provided by learners to patients/carers, and others involved with their care or service	4	2
4 I feel able to listen respectfully to the expressed needs of all parties in shaping and delivering care or services	5	1
5 I feel able to discuss patient/carer expectations or demands of antimicrobials and the need to use antimicrobials appropriately	4	2
6 I feel able to recognize patient social‐economic restrictions (or other conditions of vulnerability) that may limit the appropriate course of antimicrobials, and support patients and their families for social protection achievement	4	2
7 I feel able to recognize patients and families who require support to complete a course of antimicrobial therapy	4	2
Domain six: Interprofessional collaborative practice Competency statement: All qualified healthcare professionals need to understand how different professions collaborate in relation to how they contribute to AS.		
I feel able to demonstrate an understanding of the roles, responsibilities and competencies of other health professionals involved in antimicrobial treatment policy decisions	4	2
2 I feel able to explain why it is important that healthcare professionals, involved in the delivery of antimicrobial therapy (including the prescription, delivery and supply), have a common understanding of antimicrobial treatment policy decisions, the quantity of antimicrobial use and effective patient/client outcomes	4	2
3 I feel able to establish collaborative communication principles and actively listen to other professionals and patients/carer involved in the delivery of antimicrobial therapy	4	1
4 I feel able to communicate effectively to ensure common understanding of care decisions	4	1
5 I feel able to develop trusting relationships with patients/carer and other health/social care professionals	5	1
6 I feel able to effectively use information and communication technology to improve interprofessional patient‐centred care	5	1

The level and strength of agreement were also high for descriptors within domain three the diagnosis of infection and use of antibiotics (4 or 5 on the 5‐point Likert scale IQR ≤ 2), but these levels were lower for six descriptors (3 on the Likert scale) (see Table 2).

**TABLE 2:** Descriptors in domain three with lower levels of agreement.

‘I feel able to interpret microbiology results/reports from the laboratory’. ‘I feel able to explain why self‐limiting bacterial or viral infections are unlikely to benefit from antimicrobials’. ‘I feel able to describe broad spectrum and narrow spectrum antimicrobials and the contribution of broad spectrum antimicrobials to antimicrobial resistance’. ‘I feel able to describe what is meant by delayed prescribing’. ‘I feel able to demonstrate an awareness of laboratory results (i.e. culture and sensitivity that demand prompt intervention)’. ‘I feel able to recognize antimicrobials that should be preserved for treatment of specific infections e.g. carbapenemase‐producing Enterobacteriaceae (CPE) or colistin –resistance or colistin resistant pathogens’.

The level and strength of agreement were high (4 or 5 on the 5‐point Likert scale and an IQR ≤ 2) for competency descriptors within domain four (antimicrobial prescribing practice).

However, levels of agreement were lower for four of these descriptors (Median 3) (see Table 3).

**TABLE 3:** Descriptors in domain four with lower levels of agreement.

‘I feel able to understand the appropriateness of antimicrobial administration models such as outpatient parenteral antimicrobial therapy (OPAT)’. ‘I feel able to discuss factors that can influence antimicrobial prescribing and the implications for antimicrobial stewardship programmes’. ‘I feel able to explain how you would identify the medicines with which antimicrobials can interact and why this is important’. ‘I feel able to describe the difference between empiric, targeted and prophylactic antimicrobial therapy’.

Level and strength of agreement were high (4 or 5 on the 5‐point Likert scale and an IQR ≤ 2) for domains five and six PCC and ICP.

### 5.3 Teaching methods

There were high levels of agreement that problem‐based learning (PBL), activities in the clinical setting and face‐to‐face teaching were useful AMS teaching methods (5 on the 5‐point Likert scale) (see Table 4).

**TABLE 4:** AMS Teaching and assessment methods.

	*n* = students Indicating use of each method	Median	IQR
Teaching methods			
7 Online learning	531	3	1
8 Blended learning (classroom and online activities)	521	4	2
9 Face‐to‐face taught session	520	5	1
10 Lectures	520	4	2
11 Case studies	510	4	2
12 Student presentations	493	3	2
13 Activities in clinical settings	517	5	1
14 Problem‐based learning	515	5	2
15 Use of simulators or other virtual environments	509	4	2
16 e‐learning	526	3	1
Assessment methods			
17 Assessment essay	491	4	1
18 Objective structured clinical examination (OSCE) stations	486	4	2
19 Student presentations	486	3	2
20 Student portfolio	485	3	2
21 Short answer examination	496	4	2
22 Long answer examination questions	487	3	2
23 Multiple‐choice question examination	506	4	2

The strength of agreement was also high (IQR ≤ 2). Online learning, e‐learning and student presentations were seen as less useful (3 on the 5‐point Likert scale, IQR ≤ 2).

### 5.4 Assessment methods

There was high level and strength of agreement that assessment essays, OSCE's, short answer examination questions and MCQs (4 or 5 on the 5‐point Likert scale and an IQR ≤ 2) were useful AMS assessment methods (see Table 4). Lower levels of agreement were indicated for student presentations/portfolio and long answer examination questions (3 on the 5‐point Likert scale).

### 5.5 Interprofessional learning (IPL)

One hundred and thirty‐five students (135/523, 26%) reported that they had shared AMS teaching with students from other professions. Of these students, 129 (96%) reported that it was helpful. Freetext comments indicated that this teaching had been helpful as it provided students with a broader understanding of AMS (*n* = 38), provided an understanding of the roles of others in AMS (*n* = 7) and improved interprofessional working (*n* = 9).

## 6 DISCUSSION

### 6.1 Statement of principal findings

To the authors' knowledge, this is the first national study to assess nurses understanding and skills in the application of AMS knowledge to practice, as they transition towards professional registration. The findings represent 553 third year nursing students across 23 universities. Although there were high levels of agreement across descriptors within all domains, that students felt prepared in AMS, there were a number of descriptors, within the domains which focused on the diagnosis of infection and use of antibiotics and antimicrobial prescribing practice, where levels of agreement were lower. PBL, and activities in the clinical setting, were reported as useful teaching methods, whereas online learning, was seen as less useful.

### 6.2 Comparison with other studies

Principles from the basic sciences, are important to the pre‐registration nursing curriculum (Horiuchi‐Hirose et al., 2023; Shahzeydi et al., 2022) and nursing care delivery (Wu & Mahoney, 2022). However, it is evident from our findings that there are gaps in student nurses' knowledge of the basic sciences associated with the AMS activities in which nurses are involved. These gaps were in the domains the diagnosis of infection and use of antibiotics (domain three), and antimicrobial prescribing practice (domain four). Descriptors in which students felt less prepared, were those in which microbiology knowledge, and prescribing and its effect on AMS, is required. This aligns with work by McEwen and Burnett (2019) who reported a poor knowledge of antibiotics and a lack of understanding of AMS among pre‐registration nursing students. Our findings also concur with a recent integrative review (Gotterson et al., 2021) in which nurses were reported to be unfamiliar with the links between antimicrobial use and AMR, unfamiliar with the term AMS and had suboptimal knowledge of indications for the collection of microbiological specimens for culture. Our findings are also in line with those of a recent national cross sectional survey of UK pre‐registration nurse education programmes (Courtenay et al, 2021 in which it was reported that a greater focus on domains specifically pertaining to the use, management and monitoring of antimicrobials would help to strengthen AMS in pre‐registration programmes.

By contrast, IPC, PCC and ICP, were areas in which students reported they felt better prepared. These are areas of AMS in which nurses have previously reported that they expect to be involved. Nurses and other healthcare professionals see PCC as central to nursing practice, with nurses expected to be involved in AMS through IPC and patient education (Mostaghim et al., 2017) acting as patient advocates in de‐escalation and monitoring duration of antimicrobial therapy (Rout & Brysiewicz, 2017) and ensuring safe care. Nurses consistent presence in healthcare delivery also places them in a pivotal position for interprofessional working, that is, communicating information, such as prescribing advice (Cotta et al., 2014) to and from other health professionals, and patients. It is evident, that nurses lack clarity with regard to their role in AMS (Kirby et al., 2020). As well as IPC, PCC and ICP, AMS also comprises knowledge from the basic sciences associated with the use, management and monitoring of antimicrobials (Courtenay et al., 2019). This knowledge is essential if nurses are to be clear how they can contribute to AMS and have a positive influence on the use and management of antimicrobials.

Although nurses have reported online learning and web‐based resources to be the preferred mode of education delivery for continuing education in AMS (Fisher et al., 2018; Greendyke et al., 2018; Wilcock et al., 2019), PBL, activities in the clinical setting and face‐to‐face teaching were identified as the most useful methods by pre‐registration nursing students in our study. Interestingly, those involved in teaching AMS on UK pre‐registration nurse education programmes, report lectures as the strategy used to deliver AMS content, with PBL one of the least used methods and essays, OSCEs and MCQs reported as the methods of choice to assess nurses learning (Courtenay et al 2022). Reported benefits of IPE in AMS teaching included a broader understanding of AMS and the roles of others, and improved interprofessional working. Given that AMS is an interprofessional activity (Doron & Davidson, 2011; Fishman, 2006) and interprofessional education is an expectation of pre‐registration programmes (Health Education and Improvement Wales, 2020), the need to develop interprofessional skills is heightened.

### 6.3 Implications for policy and practice

AMS, a multidisciplinary activity, is linked to a number of nurse behaviours such as the application of fundamental infection control precautions, recognition of the signs and symptoms of infection and collaboration with the interprofessional team, to ensure appropriate antimicrobial use (Castro‐Sánchez et al., 2019; Chater et al., 2022; Courtenay et al., 2019). Although student nurses feel most prepared in IPC, PCC and ICP, that is, areas of AMS in which nurses have been reported to have a role in AMS, they feel less prepared in areas pertaining to the management of antimicrobials and specifically those areas that require knowledge from the basic sciences, that is, microbiology and prescribing. This is reflected in pre‐registration nursing programmes where knowledge from IPC, PCC and ICP takes precedent over knowledge specifically pertaining to antimicrobial management (Courtenay et al., 2021). Nurses, upon qualifying, are expected to ‘demonstrate the ability to progress to a prescribing qualification following registration’(NMC, 2018) and can access prescribing education with as little as 1 year qualified experience (NMC, 2018). Once qualified as a prescriber, nurses frequently prescribe antibiotics (Courtenay et al., 2023); therefore, there is a need to strengthen knowledge specifically pertaining to antimicrobial management in pre‐registration nurse education programmes or, ensure that postgraduate training and education in prescribing addresses the gaps in antimicrobial prescribing and management, highlighted by our study. These gaps may not only relate to the basic sciences (i.e. pharmacology and microbiology), but also the human factors (i.e. communication) that are required for optimal prescribing and antimicrobial stewardship.

Sharing AMS learning with students from other professions was reported as helpful by student nurses. Greater opportunity to study AMS, with students from other professional backgrounds (perhaps through PBL), together with exposure to multiple viewpoints about AMS, would help to strengthen nurses' knowledge of antimicrobial management and have a positive influence on interprofessional working helping to develop interprofessional teamwork skills (Nancarrow et al., 2013; Tobie et al., 2020). IPE would help other members of the AMS team understand nurses' role in AMS and vice versa, therefore nurses becoming clearer about the role they and others can play with regard to antimicrobial management. This would help nurses to become more formally recognized as part of the AMS team and more confident, likely utilizing their roles to a greater extent and maximizing their contribution to AMS.

### 6.4 Limitations

At the time of this study, the 2018 NMC Standards (NMC, 2018) had been published for 5 years. Previous standards (NMC, 2010) did not stipulate that nurses must protect health through understanding and applying AMS knowledge and skills. Data were collected from pre‐registration nursing students that were on the pre‐registration programme being phased out and the ‘updated’ programme. Therefore, data collected from students on the outgoing programme may account for some of the lack of preparedness in AMS reported by these students.

Students from 23 (32%) of the 72 universities offering pre‐registration nursing programmes in the United Kingdom took part. Although, arguably, not a representative sample, those participating were drawn from universities across all regions of the four UK countries, and universities were typical in terms of number of students recruited and academic staff employed. Furthermore, pre‐registration nursing programmes must comply with the same tightly controlled standards set by the NMC, that is, there is little scope for variation in entry requirements, clinical and academic standards, or overall teaching hours between institutions.

It was not possible to assess response rate as we do not know the total number of final year student nurses in each of the participating universities. However, the style of questions in our survey adopted forced‐response conditions (i.e. whereby participants were unable to proceed to the next question unless they respond). Therefore, all respondents completed all of the survey questions.

This study benchmarked student preparedness against a given set of competencies which, although endorsed and rigorously developed, may not be the only set of competencies to consider. Furthermore, feeling prepared, might not reflect intentions and behaviour to engage with AMS. Collecting the survey data over an extended data collection period may have influenced the findings. Those students completing the survey towards the end of the data collection period, may have felt more prepared (as compared to those participants completing the survey at the beginning of the data collection period) to engage in AMS activities as a result of experiencing more teaching in AMS.

The research topic was not a highly contentious topic, in which participants were likely to want to ‘please’ members of the research team. However, social desirability bias was reduced by assuring participants that they would remain anonymous, by avoiding the use of heavily laden questions and inviting the NAG panel to review the wording of the questionnaire.

### 6.5 Recommendations for further research

Repeating the study in nursing schools in other countries, using the established international AMS competencies used in this research, will enable continuous improvement in stewardship efforts at a global level.

## 7 CONCLUSION

There are gaps in student nurses' knowledge of the basic sciences associated with the AMS activities in which nurses are involved, and a need to strengthen knowledge in pre‐registration nurse education programmes pertaining to antimicrobial management, specifically microbiology and antimicrobial regimes and effects on AMS. IPC, PCC and ICP are areas of AMS in which students feel prepared. IPE would help nurses and other members of the AMS team clarify the role nurses can play in AMS and therefore maximize their contribution to AMS and antimicrobial management.

## FUNDING INFORMATION

This research received no specific grant from any funding agency in the public, commercial, or not‐for‐profit sectors.

Data utilized in the submitted article have been lawfully acquired in accordance with The Nagoya Protocol on Access to Genetic Resources and the Fair and Equitable Sharing of Benefits Arising from Their Utilization to the Convention on Biological Diversity.

## CONFLICT OF INTEREST STATEMENT

The authors declare no conflicts of interest.

### PEER REVIEW

The peer review history for this article is available at https://www.webofscience.com/api/gateway/wos/peer‐review/10.1111/jan.16195.

## Supporting information


Data S1:

## Data Availability

The data that support the findings of this study are available from the corresponding author upon reasonable request.
